# The construction of Chinese indicator system on public health field investigation and short-term study hub: experience and implications

**DOI:** 10.1186/s41256-022-00273-z

**Published:** 2022-10-28

**Authors:** Ning Feng, Yanhui Dong, Shelan Liu, Xiaoping Dong

**Affiliations:** 1 Center for Global Public Health, Chinese Center for Disease Control and Prevention, Room 211, 155 Changbai Road, Changping District, Beijing, People’s Republic of China; 2 Institute of Child and Adolescent Health, School of Public Health, Peking University, Beijing, People’s Republic of China; 3 Department of Infectious Diseases, Zhejiang Provincial Center for Disease Control and Prevention, Hangzhou, Zhejiang People’s Republic of China; 4 National Institute for Viral Disease Control and Prevention, Chinese Center for Disease Control and Prevention, Beijing, People’s Republic of China

**Keywords:** Indicator, Public health, Hub, Necessity, Feasibility, Coherence

## Abstract

**Background:**

The increasing of exchange activities among public health institutes and experts globally calls for a standardized operation to construct public health field investigation and short-term study hub (Field Study Hub). This can funcion as a platform to share experience in public health development in an accurate and comprehensive manner that would benefit global practices. This research aims to establish a supportive indicator system to guide the construction work.

**Methods:**

Delphi method including two rounds of surveys were conducted among 82 senior public health experts. A structured questionnaire was designed to collect the opinions of the experts on the necessity of setting and feasibility of measurement for proposed 5 dimensions of 49 indicators and 7 additionally proposed ones. Percentage and score were used to describe the assessments, χ^2^ and t tests to compare differences, Kappa and Cronbach’s alpha values to assess intra-rater and inter-rater reliabilities. Significance level α was 0.05. Bonferroni adjustment was used in the comparison of experts’ judgment basis.

**Results:**

The percentages of experts choosing “Very good” or “Good” for necessity and feasibility in rounds 1 and 2 were 73.1–97.6% (85.8% ± 7.5%), 64.6–93.9% (82.8% ± 6.7%), 73.8–100% (91.0% ± 6.2%) and 72.5–100% (89.2% ± 7.3%) respectively. The scores of necessity were higher than those of feasibility, and the differences in the dimensions of “Key experience”, “Capacity for logistic support” and the total were statistically significant (t_11_ = 2.920, t_12_ = 3.035, t_31_ = 4.448, t_32_ = 2.664, t_t1_ = 3.794, t_t2_ = 3.007, *P* < 0.05). The fourteen most necessary indicators were identified. The judgment bases of “Theory” and “Experience” were higher than “Knowledge” and “Intuition” statistically significantly (round 2: χ_TK_^2^ = 39.020, χ_EK_^2^ = 67.692, χ_TI_^2^ = 45.823, χ_EI_^2^ = 76.515, *P* < 0.0125). The Kappa values exceeded 40 with the maximum as 75 and the Cronbach’s alphas exceeded 0.8000 with the maximum as 0.9732.

**Conclusions:**

A set of 5 dimensions of 56 indicators with good necessity and feasibility were developed to technically support and well evaluate the construction of field study hub in public health institutions. This was of high significance because it tended to provide a preliminary baseline for the standardized practice in global health. Also, the present research might serve as a methodological reference for the development of other indicator sets.

**Supplementary Information:**

The online version contains supplementary material available at 10.1186/s41256-022-00273-z.

## Introduction

The promotion of global health cooperation greatly increased the exchange activities among global public health institutes and health experts [1]. Commissioners of public health in each country made persistent and tremendous efforts to strengthen public health development in order to achieve public health goals, such as Sustainable Development Goals, which brought about remarkable achievements and equipped all the masses in each country with wealth of extensive health knowledge, collaborations and experience [2–11].

Some countries played crucial roles in the development of communication mechanism and working platform in public health with public health institutes, especially for centers for disease control and prevention, and healthcare departments in women and children’s healthcare hospitals/centers at national and international levels. Such crucial role entails designing and implementing a capacity building program on global public health development cooperation [12]. In the implementation of the programs of global public health, professional public health experts were invited to different countries to have investigation visits in the centers for disease control and prevention at national, state and grass-root level. Against this background, it was popular for these programs to build qualified field study hubs inside each county’s public health system to enhance the hosting capacity of multiple levels of public health institutes, particularly in the developing countries. Such approaches would provide conducive environment for global public health experts to accurately share each country’s public health experience with international colleagues and ensure the implementation of standardized global public health practices and measures. However, few researches were conducted to identify a set of indicators pertaining to hub development for field investigation and short-term study.

According to the proposal from O’Donnell in 2020 [13], an indicator could be considered as a measure that provided an insight into relative positions in a given area or sector (e.g. public health). Evaluation of these indicators was proven to be beneficial, because it pointed out a new direction of changes in an area over a period of time and future trends [14]. Selection of suitable set of indicators relevant to establishment and evaluation of study hub entailed a high-level of judgment and consensus building among the health parastatals and health users around world [15]. However, a large number of variables might influence the development of the hub for field investigation and short-term study, hence a Delphi based-approach was necessary because a consensus might be attained amongst the public health experts. Therefore, a technical framework of indicator system to guide the development of hubs needed to be established for following reasons: the hub construction meant a considerable input of human, financial and material resources; the indicator system was expected to be a veritable tool with the capacity to circumvent possible risks from significant inputs, support to obtain satisfactory input–output ratio, accurate achievement of set goals and fully share experience after the hub’s completion.

Accordingly, the “Key experience” was set as the first dimension of the indicator system, then following four dimensions were developed as: “Capacity on experience demonstration”, “Capacity on reception”, “Capacity to host short-term study”, and “Significance of the hub construction”. Referring to professional classification of public health, international development documents such as Sustainable Development Goals [16], Agenda 2063 etc. [17] and key points were expected to be shared. We summarized China’s experience in public health into seven key areas: (1) introduction of advanced techniques to public health laboratory, (2) prevention and control of major infectious disease, (3) maternal and child healthcare, (4) disease surveillance and response, (5) public health emergency, (6) public health infrastructure, (7) prevention and control of non-communicable disease. Other indicators in the 5 dimensions were all designed through the approach of brain storming among the research group, foreign visitors and reception personnel participating in past exchange activities including field investigation and short-term study in China’s public health institutes organized by the researchers in the global public health program [12].

This research described the indicator system framework, assessed the necessity of setting and feasibility measurement of proposed indicators by invited experts and determined their intra-rater and inter-rater agreements of the experts in the two rounds of surveys.

## Method

### Study design

Delphi method including two rounds of surveys were conducted among the invited public health experts in November 2019 and from August to September 2020, respectively. A total of 82 Chinese experts from different public health workplaces including China’s national, provincial centers for disease control and prevention, women and children’s healthcare hospitals/centers/institutes, China’s universities and general hospitals were included.

### General setting

China has 23 provinces, 5 autonomous regions, and 4 municipalities (Beijing, Tianjin, Shanghai and Chongqing) and 2 special administrative regions. Each of them consists of prefectures, districts/counties, communities/townships, neighborhood committees/villages (urban/rural) respectively [18]. China has one national level center for disease control and prevention, China CDC. Each province (autonomous region, municipality), prefecture and district/county has a full government-sponsored center for disease control and prevention (CDC) which constitute China’s local triple-level CDC system consisting of 3384 CDCs in total. Besides, each province (autonomous region, municipality), prefecture and district/county has a full government-sponsored hospital/center/institute for women and children’s health (WCH) which constitute China’s local triple-level WCH system consisting of 3052 WCH institutes in total [19]. Inside China CDC, there is a National Center for Women and Children’s Health (NCWCH). China CDC plays a technical guidance role to local CDCs and WCH institutions.

The public health field investigation and short-term study hub is aimed to establish a professional exchanging platform through which the counterparts could conduct exchange visits and study activities in general or some specific areas in public health to share experience and learn from each other. In China, it is expected to be constructed in national and provincial CDCs and WCH hospitals basing on their present resources mainly with necessary enhancement. The construction of such hub also means to increase the administrative and professional functions of these institutes to receive foreign counterpart professionals and share China’s experience in public health development including concrete knowledge and skill transfer with foreign professionals through two forms of exchanging activities. One activity is field investigation through which the foreign visitors are invited to visit all levels of public health institutes for a week to understand the good practices of China in public health from various aspects. The other activity is short-term study through which the foreign participants work in these institutes to learn professional knowledge and skills for 12 weeks under the guidance of Chinese experts.

### Development of indicator framework: dimensions and indicator set

**Fig. 1:**
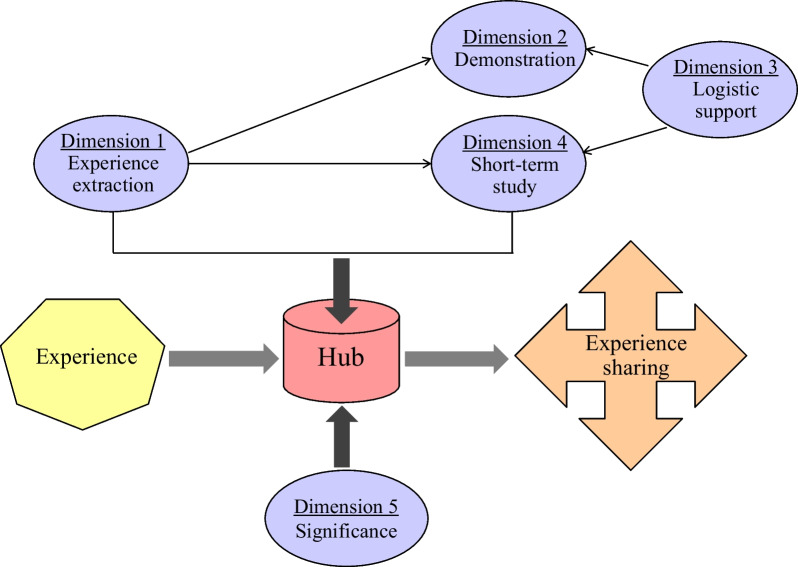
Framework the hub construction to develop the dimensions of indicator system

Since the main aim of the construction and operation of the public health field investigation and short-term study hub is to share China’s experience in public health development with foreign counterpart professionals accurately and comprehensively. For this, the first step is to extract the experience (Dimension 1), then carry out field investigation and short-term study activities with certain capacities to demonstrate and share the experiences (Dimensions 2 and 4), and logistic support (Dimension 3) is needed for the reception of the participants in field investigation and short-term study. Thus, the Dimensions 1–4 implement the functions and roles of the public health investigation and short-term study hub. In total, the public health investigation and short-term study hub will have multiple significances (Dimension 5). So, the 5 dimensions were developed according to the construction framework of the public health field investigation and short-term study hub (Fig. 1). The indicators in each dimension were generated respectively afterwards.

We adopted the Delphi method to attain expert consensus on the indicators. Consequently, a structured questionnaire (Additional file 1) was designed to assess the opinions and agreements of these experts on the degrees of necessity of setting and feasibility of measurement for proposed indicators. A five-point Likert scale including very good, good, middle, poor and very poor was used. The experts were also given an option in the questionnaire to show their judgment basis regarding theory, experience, international and domestic understanding, and intuitiveness. The judgment basis degrees were classified as high, middle and low. The Delphi process took two rounds of surveys through electronic system. Initial sets including five dimensions and their corresponding indicators were shown in Table 1. During the first round, the experts were encouraged to add indicators if they thought them necessary and feasible. These indicators were added to the pool, sorted and finalized in the second round.

**Table 1:** Dimensions one to five and their indicators

Aspect	Indicator			Type
*Dimension 1. Key experience*				
1.1 Introduction of new technique and construction of laboratory network	1.1.1 Introduction of new technique and construction of laboratory network			Open question
1.2 Prevention and control of major infectious diseases	1.2.1 Prevention and control of major infectious diseases			Open question
1.3 Maternal and child health	1.3.1 Maternal and child health			Open question
1.4 Health emergency	1.4.1 Health emergency			Open question
1.5 Construction of public health institute	1.5.1 Construction of public health institute			Open question
1.6 Prevention and control of non-communicable diseases	1.6.1 Prevention and control of non-communicable diseases			Open question
1.7 Big data and disease surveillance	1.7.1 Big data and disease surveillance			Open question
1.8 The experience summarized are correct	1.8.1 The experience summarized are correct			Ordinal variable
1.9 The experience summarized are comprehensive	1.9.1 The experience summarized are comprehensive			Ordinal variable
*Dimension 2. Capacity for experience demonstration*				
2.1The demonstration is accurate	2.1.1The demonstration is accurate			Ordinal variable
2.2 The demonstration is comprehensive	2.2.1 The demonstration is comprehensive			Ordinal variable
2.3 The demonstration has various forms	2.3.1 Has exhibition hall and posters			Ordinal variable
	2.3.2 Has PPT for introduction			Ordinal variable
	2.3.3 Has video for introduction			Ordinal variable
2.4 The rationality of the agenda	2.4.1 The rationality of the agenda			Ordinal variable
2.5 Language capability	2.5.1 Has English version at least			Ordinal variable
	2.5.2 Has personnel who can introduce in English			Ordinal variable
*Dimension 3. Capacity for logistic support*				
3.1 Human resource	3.1.1 Has special working group			Ordinal variable
	3.1.2 The division of the working group is comprehensive			Ordinal variable
	3.1.3 The working group can be mobilized timely			Ordinal variable
3.2 Reception site	3.2.1 Has reception site			Ordinal variable
3.3 Food and accommodation	3.3.1 Capacity to arrange food for visitors			Ordinal variable
	3.3.2 Capacity to arrange accommodation for visitors			Ordinal variable
3.4 Security	3.4.1Has security for visitors			Ordinal variable
3.5 Respect for the cultural identity of visitors	3.5.1 Respect for the cultural identity of visitors			Ordinal variable
*Dimension 4. Capacity for host short-term study*				
4.1 Specific technical expertise	4.1.1 Specific technical expertise			Open question
4.2 The level of specific technical expertise	4.2.1 The level of specific technical expertise			Ordinal variable
4.3 Hardware conditions for short-term study	4.3.1 Has fixed and sufficient space			Ordinal variable
	4.3.2 has necessary equipment and instruments			Ordinal variable
	4.3.3 Necessary reagent consumables are available			Ordinal variable
4.4 Software conditions for short-term study	4.4.1 Has teachers for short-term study			Ordinal variable
	4.4.2 Has short-term study plan			Ordinal variable
	4.4.3 Has research work involving short-term study visitors			Ordinal variable
	4.4.4 Short-term study visitors can continue cooperation after returning home			Ordinal variable
4.5 Description of core theory contents and class hours	4.5.1 Description of core theory contents and class hours			Open question
4.6 Description of experiment operation contents and class hours	4.6.1Description of experiment operation contents and class hours			Open question
4.7 Description of practical operation contents and class hours	4.7.1Description of practical operation contents and class hours			Open question
*Dimension 5. Significance of hub construction*				
5.1 Reception experience	5.1.1 Reception experience			Ordinal variable
5.2 Significance to host institute	5.2.1 Significance to host institute			Open question
5.3 Significance to participating individual	5.3.1 Significance to participating individual			Open question
5.4 The expectation on hub construction	5.4.1The expectation on hub construction			Open question

### Data collection

The self-administered questionnaire was distributed to the same experts in both first and second rounds of surveys. The distribution was conducted via two channels: directly through WeChat or email for the national level experts, and through office automatic (OA) system or fax from China CDC to provincial CDCs and WCH institutes who were asked to recommend experts with appropriate professional background as well as senior professional and technical title from their own institutes to participate in the evaluation. Filled forms were then returned to the researcher within 3 weeks by emails. Experts in the first round included 30 nation-level experts. The provincial CDCs and WCH hospitals recommended 52 local experts, hence a total of 82 experts responded to the questionnaire. In the second round, two local experts could not participate due to the retirement and emergency work respectively.

### Statistical analysis

The data collected was double-entered with validation using Epidata Entry version 3.1 and exported into Statistical Package for Social Science (SPSS) version 22 for data analysis. The distributions of the responses were described by number of counts (percentage, %), minimum, maximum and mean ± standard deviation (sd). Chi-square (χ^2^) test and student’s t test were used to compare the differences between necessity of setting and feasibility of measurement in rounds 1 and 2. The intra-rater and inter-rater agreements of expert judgments were assessed by Kappa and Cronbach’s alpha values respectively.

For necessity of setting and feasibility of measurement of the indicators, the scales of “Very good”, “Good”, “Middle”, “Poor” and “Very poor” corresponding to 5, 4, 3, 2 and 1 respectively. The scores were then calculated by using the formula: Score = Number of Very good*5 + Number of Good*4 + Number of Middle*3 + Number of Poor*2 + Number of Very poor*1. For the expert judgment basis, the degree of “High” valued 5, “Middle” valued 3 and “Low” valued 1. The scores were then calculated by using the formula: Score = Number of High*5 + Number of Middle*3 + Number of Low*1. Their percentages were obtained by the formula: Score*100/[82*(80 for Round 2)*5(dimensions)*5]. The indicators with higher degrees of necessity and feasibility as well as with higher agreement were considered as qualified ones. Significance level α was set to be 0.05. Bonferroni adjustment was used in the comparison of experts’ judgment basis and the adjusted α was 0.0125 (0.05/4 times of comparisons).

## Results

### Expertise areas of the experts

In the two rounds of surveys, all questionnaires were returned valid (100%), although only 2 experts were unavailable in the second round of survey. The experts that responded to the questionnaire covered 9 major expertise areas in public health (Additional file 2). Some experts are professionals in more than one area of specialization. The top 3 areas were health administration, prevention and control of major infectious diseases, and women and children’s health.

### Necessity of setting and feasibility of measurement by the experts

**Fig. 2:**
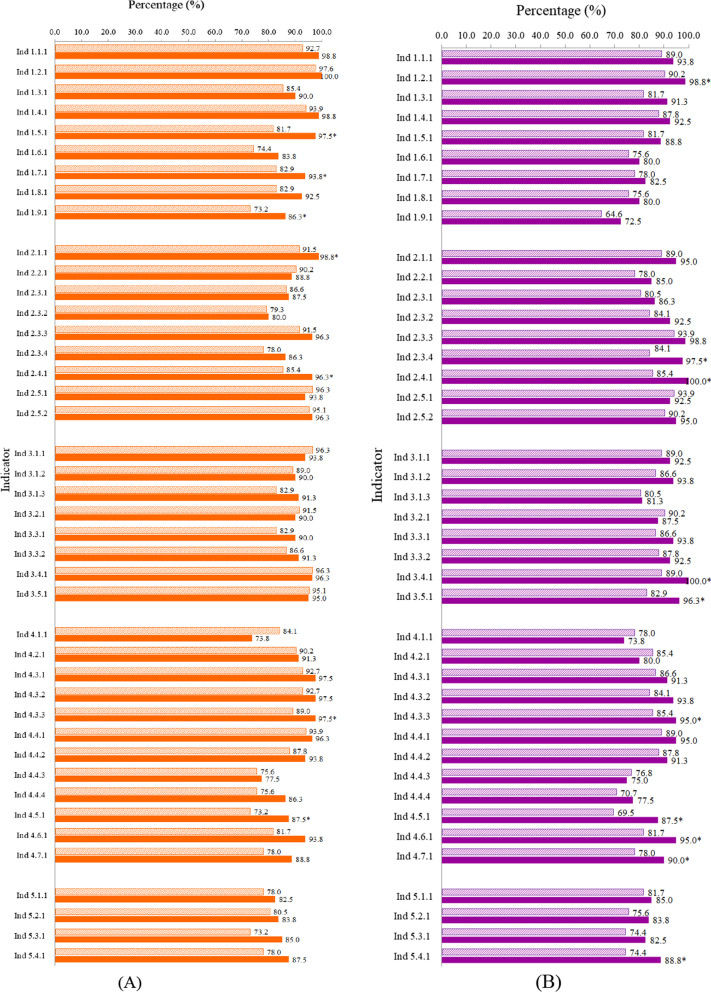
The percentages of experts who considered the necessities or feasibilities were “Very good” or “Good”. *Note* “Ind” represents “Indicator”

In both rounds, most experts agreed with the necessity of setting and feasibility of measurement of the indicators (Additional files 3). For both necessity and feasibility, the average percentages of experts who chose “Very good” or “Good” were larger than 80% with standard deviations being less than 8% totally, and the percentages in round 2 were higher than those in round 1 statistically significant (t_necessity_ = 3.443, t_feasibility_ = 4.143, *P* < 0.05) (Additional files 4). Over 85% of the indicators obtained higher ratios of “Very good” plus “Good” in the second round than in the first round, and some differences were statistically significant (*P* < 0.05) (Fig. 2A, B).

In both rounds, the scores of necessities were higher than those of the feasibilities for almost all of the indicators, and the differences in Dimension 1, Dimension 3 and the total were statistically significant (t_11_ = 2.920, t_12_ = 3.035, t_31_ = 4.448, t_32_ = 2.664, t_t1_ = 3.794, t_t2_ = 3.007, *P* < 0.05). The top indicators in necessity in round 2 achieving higher scores were: Indicators 1.2.1, 1.4.1 and 1.1.1 in Dimension 1, Indicators 2.5.1, 2.5.2 and 2.1.1 in Dimension 2, Indicators 3.5.1 and 3.4.1 in Dimension 3, the three indicators of 4.4.1, 4.3.3, 4.3.2 in Dimension 4 and Indicator 5.1.1 in Dimension 5 in order respectively (Table 2).

**Table 2:** The scores of expert judgments for the indicators in necessity and feasibility

Dimension	Indicator	Round 1				Round 2			
		Necessity	Feasibility	t value	*P* value	Necessity	Feasibility	t value	*P* value
1	1.1.1	369	340	2.920	0.010	381	348	3.035	0.008
	1.2.1	391	350			393	363		
	1.3.1	348	328			360	343		
	1.4.1	375	339			383	349		
	1.5.1	343	324			368	341		
	1.6.1	329	317			339	321		
	1.7.1	357	325			360	325		
	1.8.1	346	316			352	319		
	1.9.1	319	302			330	308		
2	2.1.1	361	333	1.921	0.073	371	348	0.260	0.798
	2.2.1	352	321			336	327		
	2.3.1	337	323			332	335		
	2.3.2	336	340			330	342		
	2.3.3	363	362			359	373		
	2.3.4	333	332			343	352		
	2.4.1	348	341			363	361		
	2.5.1	378	356			376	364		
	2.5.2	375	349			376	365		
3	3.1.1	376	345	4.448	0.001	364	351	2.664	0.019
	3.1.2	361	333			355	340		
	3.1.3	349	326			358	329		
	3.2.1	361	343			353	334		
	3.3.1	350	343			347	347		
	3.3.2	352	344			353	353		
	3.4.1	380	345			374	357		
	3.5.1	378	343			375	357		
4	4.1.1	319	310	1.913	0.069	282	269	1.686	0.106
	4.2.1	344	327			334	317		
	4.3.1	362	335			364	338		
	4.3.2	365	335			367	353		
	4.3.3	360	334			371	346		
	4.4.1	366	341			377	354		
	4.4.2	351	339			359	343		
	4.4.3	326	309			323	309		
	4.4.4	328	302			329	303		
	4.5.1	287	278			328	321		
	4.6.1	319	313			350	335		
	4.7.1	310	302			340	324		
5	5.1.1	329	332	0.174	0.867	330	329	0.557	0.598
	5.2.1	303	298			316	311		
	5.3.1	297	298			319	312		
	5.4.1	301	294			315	316		
Total		346.05	327.79	3.794	0.000	350.83	336.48	3.007	0.004

### The judgment bases of the experts

The percentages of the scores for “Theory” and “Experience” were higher than those of “Knowledge about international and domestic situation” and “Intuition” statistically significantly in both round 1 (χ_TK_^2^ = 27.617, χ_EK_^2^ = 49.377, χ_TI_^2^ = 17.329, χ_EI_^2^ = 35.261, *P* < 0.0125) and round 2 (χ_TK_^2^ = 39.020, χ_EK_^2^ = 67.692, χ_TI_^2^ = 45.823, χ_EI_^2^ = 76.515, *P* < 0.0125) (Additional file 5, Fig. 3).

**Fig. 3:**
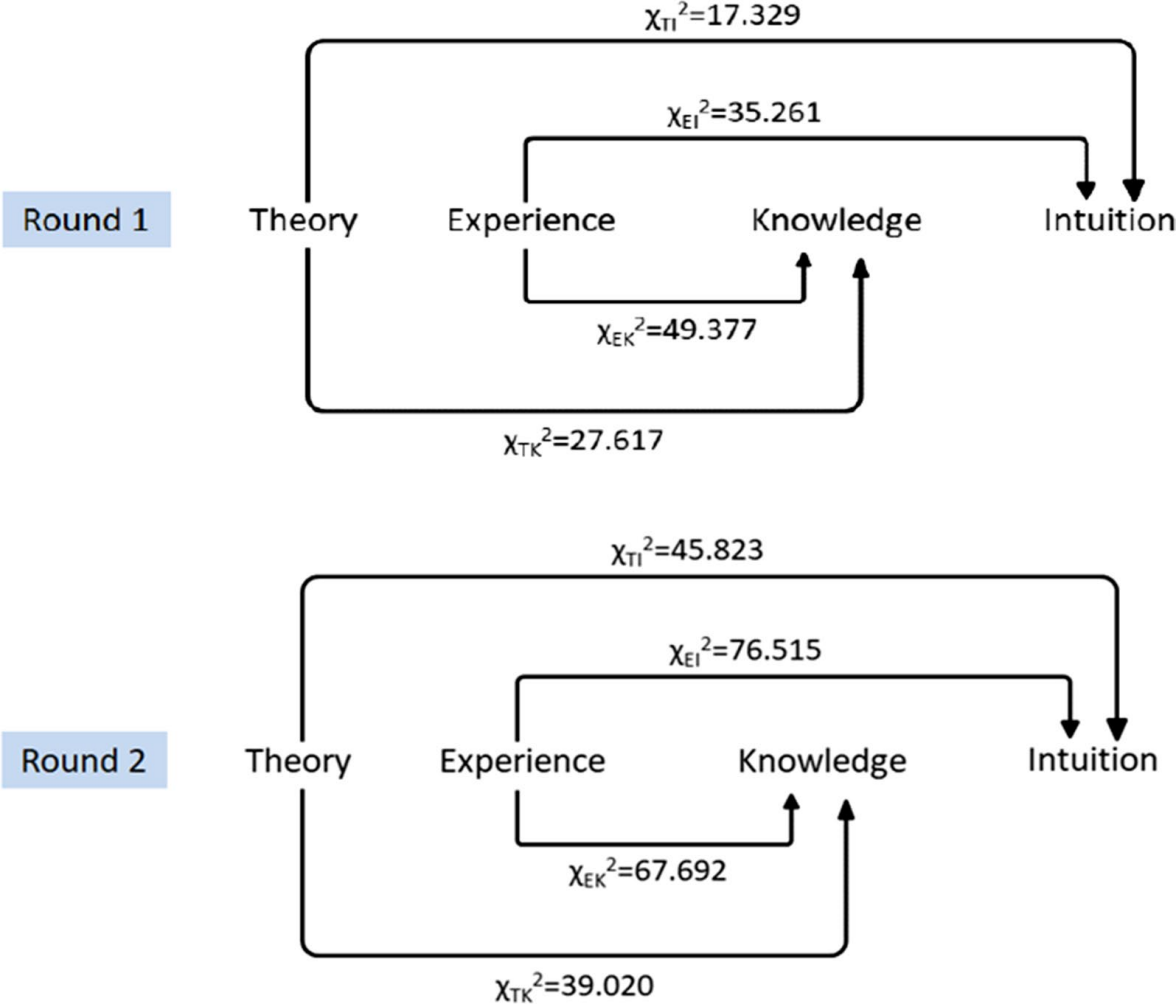
The illustration of the comparison processes of four judgment bases in round 1 and round 2, separately. *Note* *P < 0.05.

### Coherence of expert’s judgment

**Fig. 4:**
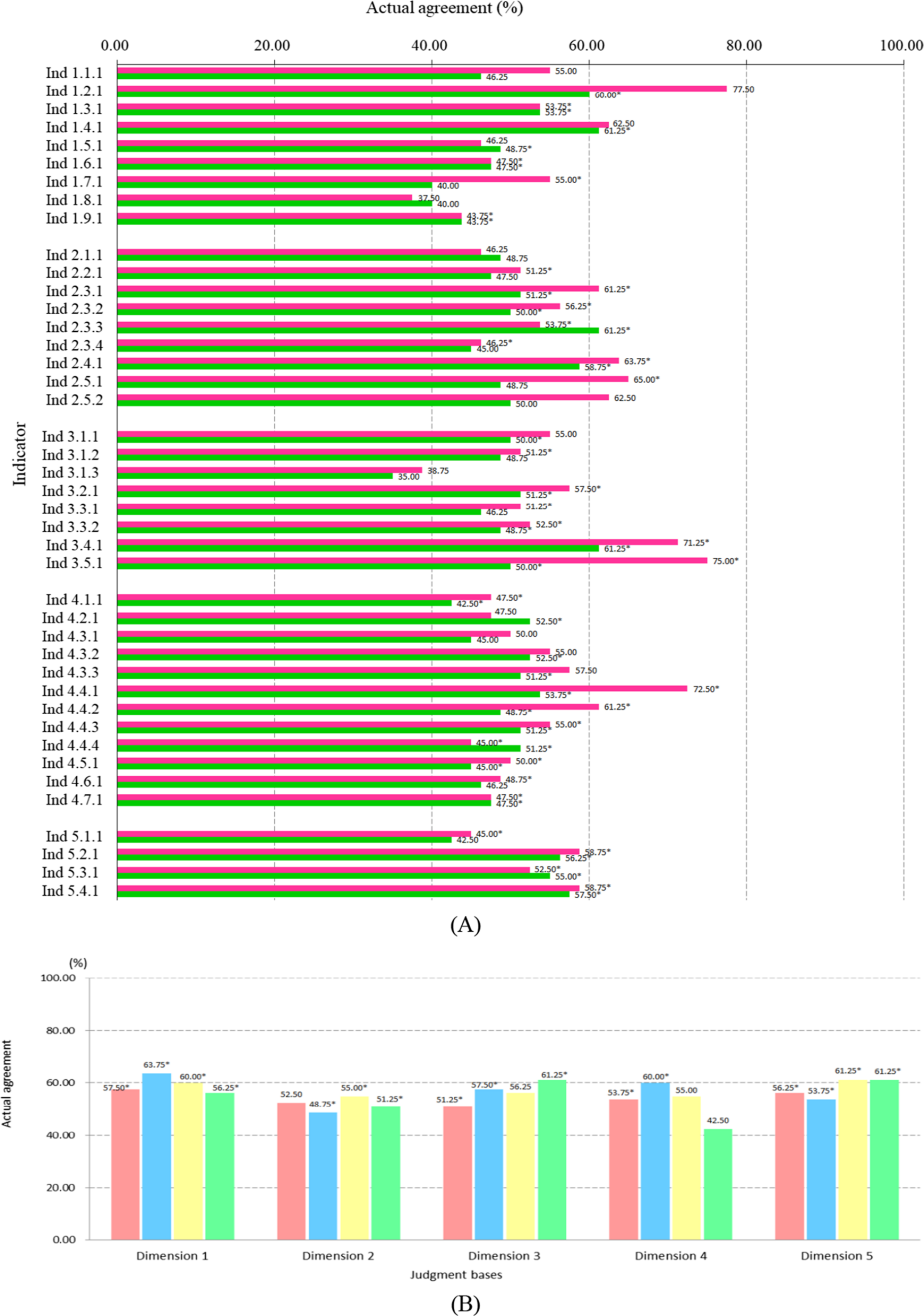
Actual agreements of Kappa values for necessity, feasibility and judgment bases between two rounds

**Fig. 5:**
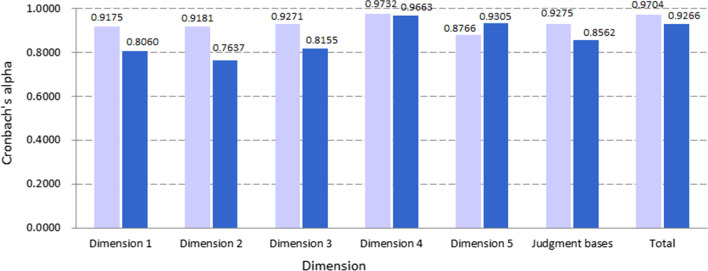
Cronbach’s alphas among experts

The Actual agreement of Kappa values of necessity and feasibility between the two rounds were 37.50–77.50 and 35.00–61.25 respectively. Twenty-eight and twenty-four indicators’ Kappa values in necessity and feasibility respectively were statistically significant (*P* < 0.05) and larger than 40.00 (*P* < 0.05), in which indicator 3.5.1’s in necessity was up to 75.00 (Fig. 4). For judgment bases, except for “Theory” in Dimensions 2 and 6 and “Knowledge about international and domestic situation” in Dimensions 3 and 4, all Kappa values were statistically significant (*P* < 0.05) and larger than 40.00 (Fig. 4B). Cronbach’s alphas demonstrated that the coherence among experts in each round of survey were larger than 0.8000 except for Dimension 2 of round 2 (0.7637). Interestingly, all Cronbach’s alphas in round 1 were larger than 0.9000 (Fig. 5).

### Additional proposed indicators by experts

Seven indicators distributed in the 5 dimensions were proposed additionally by experts in round 1 (Additional file 6). In round 2, 77.5–96.3% (90.1% ± 7.1%) and 67.6–97.6% (87.0% ± 10.8%) experts considered that the added indicators were “Very good” or “Good” in necessity and feasibility respectively (t = 0.575, *P* > 0.05) (Additional file 7).

## Discussion

The present research efficiently utilized resources and wealth of practical experience from the experts in multiple branches of public health areas from China’s both national and provincial public health institutes. Also, the selection of experts from different public health institutes and areas played a positive role to circumvent a certain degree of bias due to the same background. Through the Delphi-based approach, we obtained the necessity of setting and feasibility of measurement of 56 indicators of 5 dimensions which would form a guideline and baseline study for building an indicator system which was expected to enhance the construction of an efficient and effective public health field investigation and short-term study hub of significant scientific value in China.

In this research, we took advantage of China’s public health network in organization and mobilization during the data collection of the expert opinions, thus ensuring the participation of a wide range of experts and their opinions gained. We also emphasized on the accuracy in experience sharing with the public health professionals globally. Although there have been a few of researches on the indicator system in health area [20–22], there was a dearth of information from previous researches on the construction of hub to share public health development experience. The indicator system developed in this study is the first set of indicators to guide, evaluate and monitor the hub construction for global sharing of public health experience in China. Its whole process of the development was scientific and consistent. Attainment of a consensus tends to be an established standard for the completion of Delphi process; and capacity to reduce the variance in the outcomes is the priority for establishing the consensus [23]. Established on these virtues, this research provides a model on the methodology and procedure to the development of supportive guidelines before the actions in public health hub construction. Here, we will discuss the necessity and feasibility, coherence and future needs of the indicator system.

The judgments of the experts on both necessities of setting and feasibility of measurement of indicators were found to be different in rounds 1 and 2 of the consultations. The percentage of experts who considered necessities or feasibilities on a point scale of “Very good” or “Good” were higher in round 2 compared to round 1 consultations, and 7 and 11 indicators were statistically different (*P* < 0.05) for necessity and feasibility respectively (Fig. 2). The increased percentage observed in round 2 could be that the experts’ view on the critical value of the indicators gained more recognition. The increased Cronbach’s alphas among experts in the significance of the field investigation and short-term study hub construction for Dimension 5 validates the above rationale although the Cronbach’s alphas among experts for other dimensions were slightly lower in round 2 than in round 1 (Fig. 5).

Necessity of setting and feasibility of measurement were the two essential factors considered for the value of each indicator in this research. Interestingly, the necessity received better judgments than the feasibility for almost all of the indicators, and the differences of Dimension 1 (experience sharing), Dimension 3 (demonstration capacity) and the total were statistically significant (*P* < 0.05). While for the additional proposed indicators by experts, the necessity and feasibility values were similar. This attractive finding indicated that feasibility of indicators merited more attention when creating indicator system. The indicators should be designed specifically to get the measurement target. If necessary, the indicator should be adjusted to be more measurable and ensure a pre-test run before adoption for normal use. Meanwhile, the actual agreements of Kappa values of necessity between the two rounds were larger than those of feasibility for most indicators (Fig. 4). Perhaps the reason for this is that necessity is easier to be achieved than feasibility when designing an indicator. The statistically significant lower scores for feasibility than necessity for experience sharing and logistic support also illustrated that they are themselves relatively more difficult parts to operate besides the measurement aspect. Critical analysis of these two parts would provide a better reflection of the inherent characteristics of the public health field investigation and short-term study hub, hence more deliberations on them are essential.

Furthermore, during the round 2 survey, fourteen indicators with highest scores in necessity in their own dimensions were identified. These highlighted the following crucial points: prevention and control of major infectious diseases, health emergency and introduction of new technique, and construction of lab network were the most important areas of Chinese experience in public health; accurate content and language capacity were the most important points for experience demonstration; cross-cultural awareness and humanitarian act as well as security were important principles in reception; the importance of the sustainability of cooperation, research work and plan in short-term study were notable; and the reception experience was emphasized. For additionally proposed indicators, “Has perfect management system” (Dimension 3) and “Trainee’s evaluation on the Hub” (Dimension 5) got highest scores in necessity together with best feasibility. These fourteen indicators can be used as a group of core and short-list indicators to guide and evaluate the hub construction, especially when time is relatively tight, multiple periodic verifications on the public health field investigation and short-term study hub construction progress are needed and so on. Moreover, taken into consideration that expert opinion scores on necessity was statistically significantly higher than that of feasibility for Dimension 1 and 3 indicators as mentioned above, it is highly recommended that specific and in-depth research on the content and requirement of the six key indicators in Dimensions 1 and 3 be conducted.

Generally speaking, the Kappa value between 40 and 75 represents a middle degree of agreement, being equal or larger than 75 means a good agreement [24, 25]. The Cronbach’s alpha between 0.7 and 0.8 indicates acceptable consistency, and between 0.8 and 0.9 considerable consistency, being equal or larger than 0.9 means a very good consistency [26, 27]. The present research invited more than 80 experts from both national and provincial levels, but achieved good consistency among experts and between the two rounds of surveys, in both indicators and expert judgment basis, illustrating a good credibility of the indicators. Therefore, all initially proposed indicators were retained after the two rounds of expert consultation. The fact that the expert judgment basis was more from “Theory” and “Experience” than “Knowledge about international and domestic situation” and “Intuition” especially in the second round was consistent with the actual situation that the consulted experts might be the participants of the field investigation and short-term study hub construction and the users of the indicator system as well.

Some question items were designed as “open question”, especially for those the experience extraction in Dimension one. The responses to them are expected to be analyzed by using the method for qualitative survey materials analysis. The answers to the open questions are sorted out and analyzed to describe the responses results on the basis of the topic, the case and the case code classification. Semantic analysis of artificial intelligence could be used. For experience extraction, the resources input (human, financial and material reserves and mobilization), strategies (key points, risk avoidance) and actions (content, frequency, intensity), the significance and effect (importance, positive impact) are used as the primary (secondary) classification criteria. A method based on Bert (Bidirectional Encoder Representation from Transformers) model could be used for analysis [28, 29]. In practical operations, minor adjustment to Bert could be made according to specific downstream tasks to adapt to the text characteristics. Otherwise, we may also analyze the text about the public health experience from the perspective of topic extraction to use the author topic model for topic analysis [30]. The core content of the comment could be extracted and analyzed. In the situation where the collected responses about the experience can mostly be classified into pre-established groups, the Bert classification method can be used. When the collected responses were innovative comparatively to have more text that is difficult to be classified into the established categories, the topic analysis method will be more appropriate.

The initial purpose of present indicator system was to technically guide but not limited in evaluating the construction of the field investigation and short-term study hub in public health. However, the present research has highlighted the capacity of this indicator system to evaluate the construction of such hub objectively, which demonstrates the innovative and unique nature of present research. A step further to consolidate it may invite foreign counterparts who have been or may become participants of the field investigation and short-term study in the Hubs in China to contribute their ideas on the necessity, feasibility and supplementation of the indicators. In addition, in the course of monitoring these indicators, one could ascertain the reliability of them.

The present research would not be oblivious of certain limitation, in as much as its details were described for the experts prior to the survey, the experts’ understandings of the questionnaire might vary because the survey was carried out through email instead of face to face. Further research would be conducted with the public health experts to investigate the significance and value of each indicator. This pragmatic approach would ensure that the indicator system is a practical tool to observe the progress of the construction work about the public health field investigation and short-term study hub health supportively.

A healthy nation is a wealthy nation and the health labor force is the future driving force of the country’s overall sustainable development. With the increasing demand for standard global public health practices, the populace needs to have access to enhanced health facilities. Identification and development of these indicator sets will provide baseline for the implementation of better health strategies and healthy policies that will promote the overall capacity of public health sector including CDCs and WCH institutes in China. Furthermore, the development is helpful for the establishment, evaluation and monitoring of the hub system for field investigation and short-term study, hence meeting up with health demands now and in future using a scientific approach.

## Conclusions

A set of 5 dimensions with 56 indicators were developed to technically support and well guide a standardized construction of investigation visit and short-term study hub in public health in China. Such indicator system was found to have good necessity of setting and feasibility of measurement with good levels of agreements between two rounds of expert consultations. This was of high significance in the public health sector as the present research tended to provide a preliminary baseline for field study hub construction and evaluation in public health. Also, the set of indicators might serve as a methodological reference for the development of other indicator sets.

## Supplementary Information

**Additional file 1**. Questionnaire on the construction of Chinese indicator system of public health field investigation and short-term study Hub.

## Data Availability

All data generated or analyzed during this research are included in this published article.

## Abbreviations

CDC: Center for Disease Control and Prevention
MCH: Maternal and Child Health
China CDC: Chinese Center for Disease Control and Prevention
NCWCH: National Center for Women and Children’s Health
OA: Office automatic
WCH: Women and Children’s Health
