# Supplementary material for: The construction of Chinese indicator system on public health field investigation and short-term study hub: experience and implications
Source: Glob Health Res Policy. 2022 Oct 28;7:40. doi: 10.1186/s41256-022-00273-z (PMC9615259; doi:10.1186/s41256-022-00273-z)
Supplement: Supplementary file 1 — Additional file 1. Questionnaire on the construction of Chinese indicator system of public health field investigation and short-term study Hub. [file 41256_2022_273_MOESM1_ESM.docx]

Additional file 1

**Questionnaire on the construction of Chinese indicator system of**

**public health field investigation and short-term study Hub**

October, 2019

Dear experts:

China’s Public Health Development Assistance Capacity Building Project is carrying out the construction of public health field investigation and short-term study Hub. In order to support the construction and evaluate the operation of the field investigation and short-term study Hub from technical perspective, we are aiming to establish a set of indicators currently. Accordingly, we have developed five dimensions of the indicators (Table 1), including Key experiences, Capacity for experience demonstration, Capacity for logistic support, Capacity to host short-term study and Significance of hub construction. Please judge the necessity of setting and the feasibility of measurement for each listed indicator (the gray part is the area for you to fill). In same lieu, recommend the indicators that you consider needful to be added together with the types, meanings, and options of them. In addition, please fill in table 2 about the basis and degree of your judgment. The collected data is used for collective data analysis, and we will keep your personal information confidential. Thank you for your support!

Research Team

Your name：

Your Institution：

Your title/professional qualification:

Your major and professional area：

**Table 1 Your Judgment on the indicators**

| **Indicator** | **The type and the meaning of the indicator** | **Options of the indicator** | The **necessity** of indicator setting **(**Please draw a “**○**”in the corresponding space of the selected option.**)** | | | | | |  | The **feasibility** of indicator measurement **(**Please draw a “**○**”in the corresponding space of the selected option.**)** | | | | |
| --- | --- | --- | --- | --- | --- | --- | --- | --- | --- | --- | --- | --- | --- | --- |
|  |  |  | **Very good** | **Good** | | **Middle** | **Poor** | **Very poor** |  | **Very good** | **Good** | **Middle** | **Poor** | **Very poor** |
| **1. Key experiences** |  |  |  |  | |  |  |  |  |  |  |  |  |  |
| 1.1 Introduction of new technique and construction of laboratory network | Open question, specific experiences in this area | / |  |  | |  |  |  |  |  |  |  |  |  |
| 1.2 Prevention and control of major infectious diseases | Open question, specific experiences in this area | / |  |  | |  |  |  |  |  |  |  |  |  |
| 1.3 Maternal and child health | Open question, specific experiences in this area | / |  |  | |  |  |  |  |  |  |  |  |  |
| 1.4 Health emergency | Open question, specific experiences in this area | / |  |  | |  |  |  |  |  |  |  |  |  |
| 1.5 Construction of public health institute | Open question, specific experiences in this area | / |  |  | |  |  |  |  |  |  |  |  |  |
| 1.6 Prevention and control of non-communicable diseases | Open question, specific experiences in this area | / |  |  | |  |  |  |  |  |  |  |  |  |
| 1.7 Big data and disease surveillance | Open question, specific experiences in this area | / |  |  | |  |  |  |  |  |  |  |  |  |
| 1.8 The experiences summarized are correct | Ordinal variable, the summarized experiences is scientific, worth of publicity, with advantages and outstanding characteristics. | / |  |  | |  |  |  |  |  |  |  |  |  |
| 1.9 The experiences summarized are comprehensive | Ordinal variable, be able to summarize all kinds of experience comprehensively | (1) Very good; (2) Good; (3) Middle; (4) Poor; (5) Very poor |  | |  |  |  |  |  |  |  |  |  |  |
| 1.10 The indicators you think need to be added |  |  |  | |  |  |  |  |  |  |  |  |  |  |
| (Added indicator 1 ) | The type, meaning | Options |  | |  |  |  |  |  |  |  |  |  |  |
| **Indicator** | **The type and the meaning of the indicator** | **Options of the indicator** | The **necessity** of indicator setting **(**Please draw a “**○**”in the corresponding space of the selected option.**)** | | | | | |  | The **feasibility** of indicator measurement **(**Please draw a “**○**”in the corresponding space of the selected option.**)** | | | | |
|  |  |  | **Very good** | **Good** | | **Middle** | **Poor** | **Very poor** |  | **Very good** | **Good** | **Middle** | **Poor** | **Very poor** |
| (Added indicator 2 ) | The type, meaning | Options |  | |  |  |  |  |  |  |  |  |  |  |
| (Added indicator 3 ) | The type, meaning | Options |  | |  |  |  |  |  |  |  |  |  |  |
| **2. Capacity for experience demonstration** |  |  |  | |  |  |  |  |  |  |  |  |  |  |
| 2.1 The demonstration is accurate | Ordinal variable, the experiences can be displayed scientifically without professional mistakes; the experiences displayed are the most advanced aspects and novel | (1)Very accurate; (2) Accurate; (3)General; (4) Inaccurate; (5)Very inaccurate |  | |  |  |  |  |  |  |  |  |  |  |
| 2.2 The demonstration is comprehensive | Ordinal variable，can be a comprehensive display, no omission, the display content is multifaceted | (1)Comprehensive;（2）General；(3) Not comprehensive |  | |  |  |  |  |  |  |  |  |  |  |
| 2.3 The demonstration has various forms |  |  |  | |  |  |  |  |  |  |  |  |  |  |
| 2.3.1 Has exhibition hall and posters | Ordinal variable, refers to have good exhibition hall and new display board, with electronic screen, physical or model exhibition | (1)Has good exhibition hall and new display board; (2)Has exhibition hall and display board; (3)Has no exhibition hall or display board |  | |  |  |  |  |  |  |  |  |  |  |
| 2.3.2 Has PPT for introduction | Ordinal variable, the PPT is beautiful and can introduce the experiences accurately and comprehensively | (1)Has good PPT; (2)Has PPT; (3)Has no PPT |  | |  |  |  |  |  |  |  |  |  |  |
| 2.3.3 Has video for introduction | Ordinal variable, the video film can introduce experiences very well with good audio and video effect | (1)Has good video film;(2)Has video film;(3)Has no video film |  | |  |  |  |  |  |  |  |  |  |  |
| **Indicator** | **The type and the meaning of the indicator** | **Options of the indicator** | The **necessity** of indicator setting **(**Please draw a “**○**”in the corresponding space of the selected option.**)** | | | | | |  | The **feasibility** of indicator measurement **(**Please draw a “**○**”in the corresponding space of the selected option.**)** | | | | |
|  |  |  | **Very good** | **Good** | | **Middle** | **Poor** | **Very poor** |  | **Very good** | **Good** | **Middle** | **Poor** | **Very poor** |
| 2.4 The rationality of the agenda | Ordinal variable, the content of the agenda is rich, the time arrangement of the agenda is sufficient and compact, and the content order is reasonable | (1)Has reasonable agenda;(2)Has agenda;(3)Has no agenda |  | |  |  |  |  |  |  |  |  |  |  |
| 2.5 Language capability |  |  |  | |  |  |  |  |  |  |  |  |  |  |
| 2.5.1 Has English version at least | Ordinal variable, has English version of displayed materials, correct text, can also have French version | (1)Has good English version displayed materials and other language versions;(2) Has good English version displayed materials;(3) Has English version displayed materials; (4)Has no English or French version displayed materials |  | |  |  |  |  |  |  |  |  |  |  |
| 2.5.2 Has personnel who can introduce in English | Ordinal variable, refers to have people who can introduce them skillfully in English | (1)Has people who can introduce them skillfully in English;(2)Has people who can introduce them in English;(3)Has no people who can introduce them in English |  | |  |  |  |  |  |  |  |  |  |  |
| 2.6 The indicators you think need to be added |  |  |  | |  |  |  |  |  |  |  |  |  |  |
| (Added indicator 1 ) | The type, meaning | Options |  | |  |  |  |  |  |  |  |  |  |  |
| (Added indicator 2 ) | The type, meaning | Options |  | |  |  |  |  |  |  |  |  |  |  |
| (Added indicator 3 ) | The type, meaning | Options |  | |  |  |  |  |  |  |  |  |  |  |
| **3. Capacity for logistic support** |  |  |  | |  |  |  |  |  |  |  |  |  |  |
| 3.1 Human resource |  |  |  | |  |  |  |  |  |  |  |  |  |  |
| **Indicator** | **The type and the meaning of the indicator** | **Options of the indicator** | The **necessity** of indicator setting **(**Please draw a “**○**”in the corresponding space of the selected option.**)** | | | | | |  | The **feasibility** of indicator measurement **(**Please draw a “**○**”in the corresponding space of the selected option.**)** | | | | |
|  |  |  | **Very good** | **Good** | | **Middle** | **Poor** | **Very poor** |  | **Very good** | **Good** | **Middle** | **Poor** | **Very poor** |
| 3.1.1 Has special working group | Ordinal variable, refers to have special working group for reception | (1)Has; (2) do not have |  | |  |  |  |  |  |  |  |  |  |  |
| 3.1.2 The division of the working group is comprehensive. | Ordinal variable，has comprehensive divisions of working group including professional and logistic groups etc. | (1)Comprehensive; (2)Not comprehensive; (3)The working group has no divisions/teams |  | |  |  |  |  |  |  |  |  |  |  |
| 3.1.3 The working group can be mobilized timely | Ordinal variable, the working group can be mobilized timely according to the task | (1)The working group can be mobilized timely; (2)The working group can be mobilized;(3)It is difficult to mobilize the working group |  | |  |  |  |  |  |  |  |  |  |  |
| 3.2 Has reception site | Ordinal variable, there is sufficient space of reception, where can have meetings, organize visits, etc. | (1)Has good reception site; (2)Has reception site;(3)Has no reception site |  | |  |  |  |  |  |  |  |  |  |  |
| 3.3 Food and accommodation |  |  |  | |  |  |  |  |  |  |  |  |  |  |
| 3.3.1 Capacity to arrange food for visitors | Ordinal variable, be able to arrange appropriate meals for the visitors, the price of the food conforms to the expenditure standard. | (1)Very good capacity; (2)Good capacity; (3)Middle; (4) Poor capacity; (5)Very poor capacity |  | |  |  |  |  |  |  |  |  |  |  |
| 3.3.2 Capacity to arrange accommodation for visitors | Ordinal variable, be able to arrange comfortable and convenient accommodation for the visitors | (1)Very good capacity; (2)Good capacity; (3)Middle; (4) Poor capacity; (5)Very poor capacity |  | |  |  |  |  |  |  |  |  |  |  |
| **Indicator** | **The type and the meaning of the indicator** | **Options of the indicator** | The **necessity** of indicator setting **(**Please draw a “**○**”in the corresponding space of the selected option.**)** | | | | | |  | The **feasibility** of indicator measurement **(**Please draw a “**○**”in the corresponding space of the selected option.**)** | | | | |
|  |  |  | **Very good** | **Good** | | **Middle** | **Poor** | **Very poor** |  | **Very good** | **Good** | **Middle** | **Poor** | **Very poor** |
| 3.4 Has security for visitors | Ordinal variable, refers to has safe accommodation, traffic safety and convenient medical services | (1)Very good; (2)Good; (3)Middle; (4)Poor; (5)Very poor |  | |  |  |  |  |  |  |  |  |  |  |
| 3.5 Respect for the cultural identity of visitors | Ordinal variable, refer to respect for the cultural identity of the visitors | (1)Very good; (2)Good; (3)Middle; (4)Poor; (5)Very poor |  | |  |  |  |  |  |  |  |  |  |  |
| 3.6 The indicators you think need to be added |  |  |  | |  |  |  |  |  |  |  |  |  |  |
| (Added indicator 1 ) | The type, meaning | Options |  | |  |  |  |  |  |  |  |  |  |  |
| (Added indicator 2 ) | The type, meaning | Options |  | |  |  |  |  |  |  |  |  |  |  |
| (Added indicator 3 ) | The type, meaning | Options |  | |  |  |  |  |  |  |  |  |  |  |
| **4. Capacity for host short-term study** | Short-term study refers to 12 weeks of study and work in the department |  |  | |  |  |  |  |  |  |  |  |  |  |
| 4.1 Specific technical expertise | Open question, specific and good at skills | / |  | |  |  |  |  |  |  |  |  |  |  |
| 4.2 The level of specific technical expertise | Ordinal variable，refers to the international level, the domestic level | (1) International standard/ leading level was attained; (2) domestic leading level was attained (3) provincial level was attained ; (4) The technical expertise level was poor |  | |  |  |  |  |  |  |  |  |  |  |
| 4.3 Hardware conditions for short-term study |  |  |  | |  |  |  |  |  |  |  |  |  |  |
| 4.3.1 Has fixed and sufficient space | Ordinal variable, refers to has a fixed and sufficient space for short-term trainees, including office space, laboratory operating desk, clinical practice site, etc. | (1) Has a space with very good conditions; (2) Has a space with good conditions; (3) Has a space with general conditions; (4) Has a space with poor conditions; (5) hasn’t space |  | |  |  |  |  |  |  |  |  |  |  |
| **Indicator** | **The type and the meaning of the indicator** | **Options of the indicator** | The **necessity** of indicator setting **(**Please draw a “**○**”in the corresponding space of the selected option.**)** | | | | | |  | The **feasibility** of indicator measurement **(**Please draw a “**○**”in the corresponding space of the selected option.**)** | | | | |
|  |  |  | **Very good** | **Good** | | **Middle** | **Poor** | **Very poor** |  | **Very good** | **Good** | **Middle** | **Poor** | **Very poor** |
| 4.3.1 Has fixed and sufficient space | Ordinal variable, refers to has a fixed and sufficient space for short-term trainees, including office space, laboratory operating desk, clinical practice site, etc. | (1) Has a space with very good conditions; (2) Has a space with good conditions; (3) Has a space with general conditions; (4) Has a space with poor conditions; (5) hasn’t space |  | |  |  |  |  |  |  |  |  |  |  |
| 4.3.2 has necessary equipment and instruments | Ordinal variable, refers to have necessary equipment for short-term trainees to learn good at technology | (1) Have perfect necessary equipment; (2) have basic equipment; (3)The equipment is imperfect; (4) has no equipment |  | |  |  |  |  |  |  |  |  |  |  |
| 4.3.3 Necessary reagent consumables are available | Ordinal variable, refers to have necessary reagent consumables for short-term trainees to learn good at technology | (1) Have adequate and necessary reagent consumables; (2) have basic reagent consumables; (3)The reagent consumables are imperfect; (4) has no reagent consumables |  | |  |  |  |  |  |  |  |  |  |  |
| 4.4 availability of Software resources  for short-term study |  |  |  | |  |  |  |  |  |  |  |  |  |  |
| 4.4.1 Has teachers for short-term study | Ordinal variable, has teachers who are competent to carry out short-term training in terms of technology, language and time | (1)Has good teachers;(2) Has teachers;(3)Has no teacher |  | |  |  |  |  |  |  |  |  |  |  |
| 4.4.2 Has short-term study plan | Ordinal variable, refer to have a short-term study plan with reasonable time arrangement and rich and substantial content | (1)Has a good plan; (2)Has a plan; (3) Has no a plan |  | |  |  |  |  |  |  |  |  |  |  |
| **Indicator** | **The type and the meaning of the indicator** | **Options of the indicator** | The **necessity** of indicator setting **(**Please draw a “**○**”in the corresponding space of the selected option.**)** | | | | | |  | The **feasibility** of indicator measurement **(**Please draw a “**○**”in the corresponding space of the selected option.**)** | | | | |
|  |  |  | **Very good** | **Good** | | **Middle** | **Poor** | **Very poor** |  | **Very good** | **Good** | **Middle** | **Poor** | **Very poor** |
| 4.4.3 Has research work involving short-term study visitors | Ordinal variable, refer to have appropriate scientific research work that short-term researchers can participate in | (1)Has appropriate scientific research work that short-term researchers can participate in; (2)Has scientific research work that short-term researchers can participate in; (3)Has no scientific research work that short-term researchers can participate in |  | |  |  |  |  |  |  |  |  |  |  |
| 4.4.4 Short-term study visitors can continue cooperation after returning home | Ordinal variable, after returning to homeland, participants can continue to work with the skills learned from the short-term study and carry out cooperation with us | (1) The cooperation can be continued fully; (2) The cooperation can be continued partially; (3) The cooperation cannot be continued |  | |  |  |  |  |  |  |  |  |  |  |
| 4.5 Description of core theory contents and class hours | Open question，core theory and suitability of class study duration | / |  | |  |  |  |  |  |  |  |  |  |  |
| 4.6 Description of experiment operation contents and class hours | Open question，experiment operation contents and class hours in detail | / |  | |  |  |  |  |  |  |  |  |  |  |
| 4.7 Description of practical operation contents and class hours | Open question，practical operation contents and class hours in detail | / |  | |  |  |  |  |  |  |  |  |  |  |
| 4.8 The indicators you think need to be added |  |  |  | |  |  |  |  |  |  |  |  |  |  |
| (Added indicator 1 ) | The type, meaning | Options |  | |  |  |  |  |  |  |  |  |  |  |
| (Added indicator 2 ) | The type, meaning | Options |  | |  |  |  |  |  |  |  |  |  |  |
| (Added indicator 3 ) | The type, meaning | Options |  | |  |  |  |  |  |  |  |  |  |  |
| **Indicator** | **The type and the meaning of the indicator** | **Options of the indicator** | The **necessity** of indicator setting **(**Please draw a “**○**”in the corresponding space of the selected option.**)** | | | | | |  | The **feasibility** of an indicator measurement **(**Please draw a “**○**”in the corresponding space of the selected option.**)** | | | | |
|  |  |  | **Very good** | **Good** | | **Middle** | **Poor** | **Very poor** |  | **Very good** | **Good** | **Middle** | **Poor** | **Very poor** |
| **5. Significance of hub construction** |  |  |  | |  |  |  |  |  |  |  |  |  |  |
| 5.1 Reception experiences | Ordinal variable，actually received the visitors | (1)Has reception experiences;(2)Has no reception experiences |  | |  |  |  |  |  |  |  |  |  |  |
| 5.2 Significance to host institute | Open question | / |  | |  |  |  |  |  |  |  |  |  |  |
| 5.3 Significance to participating individual | Open question | / |  | |  |  |  |  |  |  |  |  |  |  |
| 5.4 The expectation on hub construction | Open question | / |  | |  |  |  |  |  |  |  |  |  |  |
| 5.5 The indicators you think need to be added |  |  |  | |  |  |  |  |  |  |  |  |  |  |
| (Added indicator 1 ) | The type, meaning | Options |  | |  |  |  |  |  |  |  |  |  |  |
| (Added indicator 2 ) | The type, meaning | Options |  | |  |  |  |  |  |  |  |  |  |  |
| (Added indicator 3 ) | The type, meaning | Options |  | |  |  |  |  |  |  |  |  |  |  |

**Table 2 Your judgment basis and degree on the indicators**

| Dimension | Theory | | |  | Experiences | | |  | Knowledge about international and domestic situation | | |  | Intuition | | |
| --- | --- | --- | --- | --- | --- | --- | --- | --- | --- | --- | --- | --- | --- | --- | --- |
|  | High | Middle | Low |  | High | Middle | Low |  | High | Middle | Low |  | High | Middle | Low |
| 1. Key experiences |  |  |  |  |  |  |  |  |  |  |  |  |  |  |  |
| 2. Capacity for experience demonstration |  |  |  |  |  |  |  |  |  |  |  |  |  |  |  |
| 3. Capacity for logistics support |  |  |  |  |  |  |  |  |  |  |  |  |  |  |  |
| 4. Capacity to host short-term study |  |  |  |  |  |  |  |  |  |  |  |  |  |  |  |
| 5. Significance of hub construction |  |  |  |  |  |  |  |  |  |  |  |  |  |  |  |

**(**Please draw a “**○**” in the corresponding space of the selected option.**)**

Additional file 2

**Table 1.** Distribution of expertise area in public health among the experts

| **No.** | **Expertise area** | **National** | |  | **Local** | |  | **Total** | |
| --- | --- | --- | --- | --- | --- | --- | --- | --- | --- |
|  |  | **R1 (30)** | **R2 (30)** |  | **R1 (52)** | **R2 (50)** |  | **R1 (82)** | **R2 (80)** |
| 1 | Introduction of new technique and construction of laboratory network | 2 | 2 |  | 4 | 4 |  | 6 | 6 |
| 2 | Prevention and control of major infectious diseases | 3 | 3 |  | 16 | 16 |  | 19 | 19 |
| 3 | Maternal and child health | 9 | 9 |  | 2 | 2 |  | 11 | 11 |
| 4 | Health emergency | 3 | 3 |  | 6 | 6 |  | 9 | 9 |
| 5 | Prevention and control of non-communicable diseases | 2 | 2 |  | 2 | 2 |  | 4 | 4 |
| 6 | Big data and disease surveillance | 0 | 0 |  | 3 | 3 |  | 3 | 3 |
| 7 | Global health | 3 | 3 |  | 0 | 0 |  | 3 | 3 |
| 8 | Environmental health | 1 | 1 |  | 2 | 2 |  | 3 | 3 |
| 9 | Health administration | 15 | 15 |  | 18 | 16 |  | 33 | 31 |

**Additional file 3**

**Table 2 The experts’ judgment on proposed indicators (number, %)**

| **Indicator code** | **Round** | **Necessity** | | | | |  | **Feasibility** | | | | |
| --- | --- | --- | --- | --- | --- | --- | --- | --- | --- | --- | --- | --- |
|  |  | **Very good** | **Good** | **Middle** | **Poor** | **Very poor** |  | **Very good** | **Good** | **Middle** | **Poor** | **Very poor** |
| 1.1.1 | 1 | 50(61.0) | 26(31.7) | 5(6.1) | 0(0.0) | 0(0.0) |  | 34(41.5) | 39(47.6) | 4(4.9) | 1(1.2) | 0(0.0) |
|  | 2 | 62(77.5) | 17(21.3) | 1(1.3) | 0(0.0) | 0(0.0) |  | 34(42.5) | 41(51.3) | 4(5.0) | 1(1.3) | 0(0.0) |
| 1.2.1 | 1 | 68(82.9) | 12(14.6) | 1(1.2) | 0(0.0) | 0(0.0) |  | 42(51.2) | 32(39.0) | 4(4.9) | 0(0.0) | 0(0.0) |
|  | 2 | 73(91.3) | 7(08.8) | 0(0.0) | 0(0.0) | 0(0.0) |  | 44(55.0) | 35(43.8) | 1(1.3) | 0(0.0) | 0(0.0) |
| 1.3.1 | 1 | 41(50.0) | 29(35.4) | 9(11.0) | 0(0.0) | 0(0.0) |  | 33(40.2) | 34(41.5) | 9(11.0) | 0(0.0) | 0(0.0) |
|  | 2 | 48(60.0) | 24(30.0) | 8(10.0) | 0(0.0) | 0(0.0) |  | 30(37.5) | 43(53.8) | 7(8.8) | 0(0.0) | 0(0.0) |
| 1.4.1 | 1 | 58(70.7) | 19(23.2) | 3(3.7) | 0(0.0) | 0(0.0) |  | 33(40.2) | 39(47.6) | 6(7.3) | 0(0.0) | 0(0.0) |
|  | 2 | 64(80.0) | 15(18.8) | 1(1.3) | 0(0.0) | 0(0.0) |  | 35(43.8) | 39(48.8) | 6(7.5) | 0(0.0) | 0(0.0) |
| 1.5.1 | 1 | 43(52.4) | 24(29.3) | 10(12.2) | 0(0.0) | 2(2.4) |  | 32(39.0) | 35(42.7) | 6(7.3) | 2(2.4) | 2(2.4) |
|  | 2 | 50(62.5) | 28(35.0) | 2(2.5) | 0(0.0) | 0(0.0) |  | 31(38.8) | 40(50.0) | 8(10.0) | 1(1.3) | 0(0.0) |
| 1.6.1 | 1 | 31(37.8) | 30(36.6) | 18(22.0) | 0(0.0) | 0(0.0) |  | 25(30.5) | 37(45.1) | 14(17.1) | 1(1.2) | 0(0.0) |
|  | 2 | 32(40.0) | 35(43.8) | 13(16.3) | 0(0.0) | 0(0.0) |  | 21(26.3) | 43(53.8) | 12(15.0) | 4(5.0) | 0(0.0) |
| 1.7.1 | 1 | 47(57.3) | 21(25.6) | 12(14.6) | 1(1.2) | 0(0.0) |  | 28(34.1) | 36(43.9) | 11(13.4) | 4(4.9) | 0(0.0) |
|  | 2 | 46(57.5) | 29(36.3) | 4(5.0) | 1(1.3) | 0(0.0) |  | 23(28.8) | 43(53.8) | 10(12.5) | 4(5.0) | 0(0.0) |
| 1.8.1 | 1 | 39(47.6) | 29(35.4) | 9(11.0) | 4(4.9) | 0(0.0) |  | 23(28.0) | 39(47.6) | 11(13.4) | 6(7.3) | 0(0.0) |
|  | 2 | 42(52.5) | 32(40.0) | 4(5.0) | 1(1.3) | 0(0.0) |  | 19(23.8) | 45(56.3) | 14(17.5) | 1(1.3) | 0(0.0) |
| 1.9.1 | 1 | 24(29.3) | 36(43.9) | 14(17.1) | 6(7.3) | 1(1.2) |  | 15(18.3) | 38(46.3) | 22(26.8) | 4(4.9) | 1(1.2) |
|  | 2 | 23(28.8) | 46(57.5) | 10(12.5) | 0(0.0) | 1(1.3) |  | 16(20.0) | 42(52.5) | 19(23.8) | 1(1.3) | 1(1.3) |
| 2.1.1 | 1 | 52(63.4) | 23(28.0) | 3(3.7) | 0(0.0) | 0(0.0) |  | 29(35.4) | 44(53.7) | 4(04.9) | 0(0.0) | 0(0.0) |
|  | 2 | 52(65.0) | 27(33.8) | 1(01.3) | 0(0.0) | 0(0.0) |  | 32(40.0) | 44(55.0) | 4(05.0) | 0(0.0) | 0(0.0) |
| 2.2.1 | 1 | 35(42.7) | 39(47.6) | 7(8.5) | 0(0.0) | 0(0.0) |  | 20(24.4) | 44(53.7) | 15(18.3) | 0(0.0) | 0(0.0) |
|  | 2 | 26(32.5) | 45(56.3) | 8(10.0) | 1(1.3) | 0(0.0) |  | 20(25.0) | 48(60.0) | 11(13.8) | 1(1.3) | 0(0.0) |
| 2.3.1 | 1 | 29(35.4) | 42(51.2) | 6(7.3) | 3(3.7) | 0(0.0) |  | 24(29.3) | 42(51.2) | 11(13.4) | 1(1.2) | 0(0.0) |
|  | 2 | 23(28.8) | 47(58.8) | 9(11.3) | 1(1.3) | 0(0.0) |  | 26(32.5) | 43(53.8) | 11(13.8) | 0(0.0) | 0(0.0) |
| 2.3.2 | 1 | 31(37.8) | 34(41.5) | 13(15.9) | 3(3.7) | 0(0.0) |  | 34(41.5) | 35(42.7) | 10(12.2) | 0(0.0) | 0(0.0) |
|  | 2 | 27(33.8) | 37(46.3) | 15(18.8) | 1(1.3) | 0(0.0) |  | 31(38.8) | 43(53.8) | 5(06.3) | 0(0.0) | 0(0.0) |
| 2.3.3 | 1 | 46(56.1) | 29(35.4) | 5(6.1) | 1(1.2) | 0(0.0) |  | 48(58.5) | 29(35.4) | 2(02.4) | 0(0.0) | 0(0.0) |
|  | 2 | 42(52.5) | 35(43.8) | 3(03.8) | 0(0.0) | 0(0.0) |  | 54(67.5) | 25(31.3) | 1(01.3) | 0(0.0) | 0(0.0) |
| 2.3.4 | 1 | 30(36.6) | 34(41.5) | 15(18.3) | 1(1.2) | 0(0.0) |  | 29(35.4) | 40(48.8) | 9(11.0) | 0(0.0) | 0(0.0) |
|  | 2 | 34(42.5) | 35(43.8) | 11(13.8) | 0(0.0) | 0(0.0) |  | 34(42.5) | 44(55.0) | 2(02.5) | 0(0.0) | 0(0.0) |
| 2.4.1 | 1 | 38(46.3) | 32(39.0) | 10(12.2) | 0(0.0) | 0(0.0) |  | 37(45.1) | 33(40.2) | 8(09.8) | 0(0.0) | 0(0.0) |
|  | 2 | 46(57.5) | 31(38.8) | 3(03.8) | 0(0.0) | 0(0.0) |  | 41(51.3) | 39(48.8) | 0(00.0) | 0(0.0) | 0(0.0) |
| 2.5.1 | 1 | 56(68.3) | 23(28.0) | 2(2.4) | 0(0.0) | 0(0.0) |  | 42(51.2) | 35(42.7) | 2(02.4) | 0(0.0) | 0(0.0) |
|  | 2 | 64(80.0) | 11(13.8) | 4(05.0) | 0(0.0) | 0(0.0) |  | 53(66.3) | 21(26.3) | 5(06.3) | 0(0.0) | 0(0.0) |
| 2.5.2 | 1 | 57(69.5) | 21(25.6) | 2(2.4) | 0(0.0) | 0(0.0) |  | 41(50.0) | 33(40.2) | 4(04.9) | 0(0.0) | 0(0.0) |
|  | 2 | 62(77.5) | 15(18.8) | 2(02.5) | 0(0.0) | 0(0.0) |  | 52(65.0) | 24(30.0) | 3(03.8) | 0(0.0) | 0(0.0) |
| 3.1.1 | 1 | 54(65.9) | 25(30.5) | 2(2.4) | 0(0.0) | 0(0.0) |  | 36(43.9) | 37(45.1) | 5(06.1) | 1(1.2) | 0(0.0) |
|  | 2 | 49(61.3) | 26(32.5) | 5(6.3) | 0(0.0) | 0(0.0) |  | 40(50.0) | 34(42.5) | 5(6.3) | 0(0.0) | 0(0.0) |
| 3.1.2 | 1 | 45(54.9) | 28(34.1) | 8(9.8) | 0(0.0) | 0(0.0) |  | 29(35.4) | 42(51.2) | 4(04.9) | 4(4.9) | 0(0.0) |
|  | 2 | 43(53.8) | 29(36.3) | 8(10.0) | 0(0.0) | 0(0.0) |  | 26(32.5) | 49(61.3) | 4(5.0) | 1(1.3) | 0(0.0) |
| 3.1.3 | 1 | 39(47.6) | 29(35.4) | 12(14.6) | 1(1.2) | 0(0.0) |  | 25(30.5) | 41(50.0) | 11(13.4) | 2(2.4) | 0(0.0) |
|  | 2 | 45(56.3) | 28(35.0) | 7(8.8) | 0(0.0) | 0(0.0) |  | 26(32.5) | 39(48.8) | 13(16.3) | 2(2.5) | 0(0.0) |
| 3.2.1 | 1 | 44(53.7) | 31(37.8) | 5(6.1) | 1(1.2) | 0(0.0) |  | 30(36.6) | 44(53.7) | 5(06.1) | 1(1.2) | 0(0.0) |
|  | 2 | 42(52.5) | 30(37.5) | 7(8.8) | 1(1.3) | 0(0.0) |  | 24(30.0) | 46(57.5) | 10(12.5) | 0(0.0) | 0(0.0) |
| 3.3.1 | 1 | 44(53.7) | 24(29.3) | 8(9.8) | 5(6.1) | 0(0.0) |  | 32(39.0) | 39(47.6) | 9(11.0) | 0(0.0) | 0(0.0) |
|  | 2 | 38(47.5) | 34(42.5) | 7(8.8) | 0(0.0) | 0(0.0) |  | 35(43.8) | 40(50.0) | 4(5.0) | 0(0.0) | 0(0.0) |
| 3.3.2 | 1 | 44(53.7) | 27(32.9) | 4(4.9) | 6(7.3) | 0(0.0) |  | 36(43.9) | 36(43.9) | 6(07.3) | 1(1.2) | 0(0.0) |
|  | 2 | 40(50.0) | 33(41.3) | 7(8.8) | 0(0.0) | 0(0.0) |  | 39(48.8) | 35(43.8) | 6(7.5) | 0(0.0) | 0(0.0) |
| 3.4.1 | 1 | 58(70.7) | 21(25.6) | 2(2.4) | 0(0.0) | 0(0.0) |  | 36(43.9) | 37(45.1) | 5(06.1) | 1(1.2) | 0(0.0) |
|  | 2 | 57(71.3) | 20(25.0) | 3(3.8) | 0(0.0) | 0(0.0) |  | 37(46.3) | 43(53.8) | 0(0.0) | 0(0.0) | 0(0.0) |
| 3.5.1 | 1 | 57(69.5) | 21(25.6) | 3(3.7) | 0(0.0) | 0(0.0) |  | 38(46.3) | 30(36.6) | 11(13.4) | 0(0.0) | 0(0.0) |
|  | 2 | 59(73.8) | 17(21.3) | 4(5.0) | 0(0.0) | 0(0.0) |  | 40(50.0) | 37(46.3) | 3(3.8) | 0(0.0) | 0(0.0) |
| 4.1.1 | 1 | 34(41.5) | 35(42.7) | 3(3.7) | 0(0.0) | 0(0.0) |  | 30(36.6) | 34(41.5) | 8(09.8) | 0(0.0) | 0(0.0) |
|  | 2 | 32(40.0) | 27(33.8) | 4(5.0) | 1(1.3) | 0(0.0) |  | 21(26.3) | 38(47.5) | 4(5.0) | 0(0.0) | 0(0.0) |
| 4.2.1 | 1 | 39(47.6) | 35(42.7) | 3(3.7) | 0(0.0) | 0(0.0) |  | 29(35.4) | 41(50.0) | 6(07.3) | 0(0.0) | 0(0.0) |
|  | 2 | 30(37.5) | 43(53.8) | 4(5.0) | 0(0.0) | 0(0.0) |  | 23(28.8) | 41(51.3) | 12(15.0) | 1(1.3) | 0(0.0) |
| 4.3.1 | 1 | 52(63.4) | 24(29.3) | 2(2.4) | 0(0.0) | 0(0.0) |  | 33(40.2) | 38(46.3) | 6(07.3) | 0(0.0) | 0(0.0) |
|  | 2 | 52(65.0) | 26(32.5) | 0(0.0) | 0(0.0) | 0(0.0) |  | 31(38.8) | 42(52.5) | 5(6.3) | 0(0.0) | 0(0.0) |
| 4.3.2 | 1 | 55(67.1) | 21(25.6) | 2(2.4) | 0(0.0) | 0(0.0) |  | 36(43.9) | 33(40.2) | 7(08.5) | 1(1.2) | 0(0.0) |
|  | 2 | 55(68.8) | 23(28.8) | 0(0.0) | 0(0.0) | 0(0.0) |  | 44(55.0) | 31(38.8) | 3(3.8) | 0(0.0) | 0(0.0) |
| 4.3.3 | 1 | 54(65.9) | 19(23.2) | 4(4.9) | 1(1.2) | 0(0.0) |  | 34(41.5) | 36(43.9) | 6(7.3) | 1(1.2) | 0(0.0) |
|  | 2 | 59(73.8) | 19(23.8) | 0(0.0) | 0(0.0) | 0(0.0) |  | 39(48.8) | 37(46.3) | 1(1.3) | 0(0.0) | 0(0.0) |
| 4.4.1 | 1 | 55(67.1) | 22(26.8) | 1(1.2) | 0(0.0) | 0(0.0) |  | 38(46.3) | 35(42.7) | 3(3.7) | 1(1.2) | 0(0.0) |
|  | 2 | 66(82.5) | 11(13.8) | 1(1.3) | 0(0.0) | 0(0.0) |  | 45(56.3) | 31(38.8) | 1(1.3) | 1(1.3) | 0(0.0) |
| 4.4.2 | 1 | 46(56.1) | 26(31.7) | 5(6.1) | 1(1.2) | 0(0.0) |  | 37(45.1) | 35(42.7) | 4(4.9) | 1(1.2) | 0(0.0) |
|  | 2 | 53(66.3) | 22(27.5) | 2(2.5) | 0(0.0) | 0(0.0) |  | 42(52.5) | 31(38.8) | 3(3.8) | 0(0.0) | 0(0.0) |
| 4.4.3 | 1 | 32(39.0) | 30(36.6) | 14(17.1) | 2(2.4) | 0(0.0) |  | 19(23.2) | 44(53.7) | 10(12.2) | 4(4.9) | 0(0.0) |
|  | 2 | 27(33.8) | 35(43.8) | 16(20.0) | 0(0.0) | 0(0.0) |  | 20(25.0) | 40(50.0) | 13(16.3) | 5(6.3) | 0(0.0) |
| 4.4.4 | 1 | 32(39.0) | 30(36.6) | 16(19.5) | 0(0.0) | 0(0.0) |  | 17(20.7) | 41(50.0) | 15(18.3) | 4(4.9) | 0(0.0) |
|  | 2 | 26(32.5) | 43(53.8) | 9(11.3) | 0(0.0) | 0(0.0) |  | 11(13.8) | 51(63.8) | 12(15.0) | 4(5.0) | 0(0.0) |
| 4.5.1 | 1 | 27(32.9) | 33(40.2) | 6(7.3) | 1(1.2) | 0(0.0) |  | 23(28.0) | 34(41.5) | 9(11.0) | 0(0.0) | 0(0.0) |
|  | 2 | 30(37.5) | 40(50.0) | 6(7.5) | 0(0.0) | 0(0.0) |  | 23(28.8) | 47(58.8) | 6(7.5) | 0(0.0) | 0(0.0) |
| 4.6.1 | 1 | 36(43.9) | 31(37.8) | 3(3.7) | 3(3.7) | 0(0.0) |  | 30(36.6) | 37(45.1) | 5(6.1) | 0(0.0) | 0(0.0) |
|  | 2 | 41(51.3) | 34(42.5) | 3(3.8) | 0(0.0) | 0(0.0) |  | 25(31.3) | 51(63.8) | 2(2.5) | 0(0.0) | 0(0.0) |
| 4.7.1 | 1 | 36(43.9) | 28(34.1) | 4(4.9) | 3(3.7) | 0(0.0) |  | 31(37.8) | 33(40.2) | 5(6.1) | 0(0.0) | 0(0.0) |
|  | 2 | 39(48.8) | 32(40.0) | 5(6.3) | 1(1.3) | 0(0.0) |  | 21(26.3) | 51(63.8) | 5(6.3) | 0(0.0) | 0(0.0) |
| 5.1.1 | 1 | 29(35.4) | 35(42.7) | 14(17.1) | 1(1.2) | 0(0.0) |  | 31(37.8) | 36(43.9) | 11(13.4) | 0(0.0) | 0(0.0) |
|  | 2 | 25(31.3) | 41(51.3) | 13(16.3) | 1(1.3) | 0(0.0) |  | 21(26.3) | 47(58.8) | 12(15.0) | 0(0.0) | 0(0.0) |
| 5.2.1 | 1 | 16(19.5) | 50(61.0) | 6(7.3) | 2(2.4) | 1(1.2) |  | 15(18.3) | 47(57.3) | 11(13.4) | 1(1.2) | 0(0.0) |
|  | 2 | 18(22.5) | 49(61.3) | 10(12.5) | 0(0.0) | 0(0.0) |  | 13(16.3) | 54(67.5) | 10(12.5) | 0(0.0) | 0(0.0) |
| 5.3.1 | 1 | 16(19.5) | 44(53.7) | 11(13.4) | 4(4.9) | 0(0.0) |  | 16(19.5) | 45(54.9) | 12(14.6) | 1(1.2) | 0(0.0) |
|  | 2 | 20(25.0) | 48(60.0) | 9(11.3) | 0(0.0) | 0(0.0) |  | 15(18.8) | 51(63.8) | 11(13.8) | 0(0.0) | 0(0.0) |
| 5.4.1 | 1 | 20(24.4) | 44(53.7) | 8(09.8) | 0(0.0) | 1(1.2) |  | 18(22.0) | 43(52.4) | 10(12.2) | 1(1.2) | 0(0.0) |
|  | 2 | 15(18.8) | 55(68.8) | 6(07.5) | 1(1.3) | 0(0.0) |  | 14(17.5) | 57(71.3) | 6(7.5) | 0(0.0) | 0(0.0) |

**Additional file 4**

**Table 3.** The percentages of experts who chose “Very good” or “Good” for the indicators

| Dimension | Round 1 | | |  | Round 2 | |
| --- | --- | --- | --- | --- | --- | --- |
|  | Necessity  (min~max, mean±sd)  % | Feasibility  (min~max, mean±sd)  % |  | | Necessity  (min~max, mean±sd)  % | Feasibility  (min~max, mean±sd)  % |
| 1 | 73.1~97.6 (85.0±8.4) | 64.6~90.2 (80.5±8.4) |  | | 83.8~100.0 (93.5±5.9) | 72.5~98.8 (86.7±8.4) |
| 2 | 78.0~96.3 (88.2±6.5) | 78.0~93.9 (86.6±5.6) |  | | 80.0~98.8 (91.5±6.2) | 85.0~100.0 (93.6±6.0) |
| 3 | 82.9~96.3 (90.1±6.1) | 80.5~90.2 (86.6±3.9) |  | | 90.0~96.3 (92.2±2.5) | 81.3~100.0 (92.2±5.7) |
| 4 | 73.2~92.7 (84.6±7.5) | 70.7~89.0 (81.1±6.5) |  | | 73.8~97.5 (90.1±7.9) | 73.8~95.0 (87.1±8.2) |
| 5 | 73.2~80.5 (77.4±3.0) | 74.4~81.7 (76.5±3.5) |  | | 82.5~87.5 (84.7±2.1) | 82.5~88.8 (85.0±2.7) |
| Total | 73.1~97.6 (85.8±7.5) | 64.6~93.9 (82.8±6.7) |  | | 73.8~100 (91.0±6.2) | 72.5~100 (89.2±7.3) |

Additional file 5

**Table 4.** The judge basis of experts on the proposed indicators

| **Dimension** | **Round** | **Theory** | | | |  | **Experiences** | | | |  | **Knowledge about international and domestic situation** | | | |  | **Intuition** | | | |
| --- | --- | --- | --- | --- | --- | --- | --- | --- | --- | --- | --- | --- | --- | --- | --- | --- | --- | --- | --- | --- |
|  |  | **High** | **Middle** | **Low** | **Score** |  | **High** | **Middle** | **Low** | **Score** |  | **High** | **Middle** | **Low** | **Score** |  | **High** | **Middle** | **Low** | **Score** |
| One | 1 | 32 | 44 | 6 | 298 |  | 35 | 40 | 7 | 302 |  | 19 | 48 | 15 | 254 |  | 23 | 47 | 12 | 268 |
|  | 2 | 38 | 38 | 3 | 307 |  | 47 | 29 | 4 | 326 |  | 20 | 51 | 9 | 262 |  | 27 | 38 | 15 | 264 |
| Two | 1 | 29 | 45 | 8 | 288 |  | 42 | 28 | 12 | 306 |  | 19 | 47 | 16 | 252 |  | 16 | 52 | 14 | 250 |
|  | 2 | 35 | 42 | 2 | 303 |  | 43 | 33 | 4 | 318 |  | 20 | 55 | 5 | 270 |  | 21 | 44 | 15 | 252 |
| Three | 1 | 20 | 47 | 15 | 256 |  | 33 | 40 | 9 | 294 |  | 20 | 45 | 17 | 252 |  | 19 | 49 | 14 | 256 |
|  | 2 | 26 | 48 | 5 | 279 |  | 37 | 38 | 5 | 304 |  | 17 | 51 | 12 | 250 |  | 19 | 47 | 14 | 250 |
| Four | 1 | 35 | 34 | 13 | 290 |  | 31 | 38 | 13 | 282 |  | 18 | 47 | 17 | 248 |  | 23 | 43 | 16 | 260 |
|  | 2 | 36 | 33 | 7 | 286 |  | 35 | 36 | 6 | 289 |  | 20 | 49 | 8 | 255 |  | 26 | 34 | 17 | 249 |
| Five | 1 | 30 | 44 | 8 | 290 |  | 32 | 40 | 10 | 290 |  | 19 | 49 | 14 | 256 |  | 19 | 52 | 11 | 262 |
|  | 2 | 34 | 39 | 6 | 293 |  | 32 | 39 | 9 | 286 |  | 13 | 58 | 9 | 248 |  | 20 | 47 | 13 | 254 |
| Total | 1 | 146 | 214 | 50 | 1422 |  | 173 | 186 | 51 | 1474 |  | 95 | 236 | 79 | 1262 |  | 100 | 243 | 67 | 1296 |
|  | (%) |  |  |  | 69.4^*#^ |  |  |  |  | 71.9*^#^ |  |  |  |  | 61.6 |  |  |  |  | 63.2 |
|  | 2 | 169 | 200 | 23 | 1468 |  | 194 | 175 | 28 | 1523 |  | 90 | 264 | 43 | 1285 |  | 113 | 210 | 74 | 1269 |
|  | (%) |  |  |  | 73.4^*#^ |  |  |  |  | 76.2^*#^ |  |  |  |  | 64.3 |  |  |  |  | 63.4 |

Notes:

^*^ *P*<0.05, Theory base or Experience base vs Knowledge about international and domestic situation base.

^#^ *P*<0.05, Theory base or Experience base vs Intuition base

**Additional file 6**

**Table 5.** Additional proposed indicators by experts

| **Dimension** | **Indicator** | **Type** | **Code** |
| --- | --- | --- | --- |
| One | 1) The experiences are sustainable | Ordinal question | 1.10.1 |
| Two | 1)  Has the exhibition for field work | Ordinal question | 2.6.1 |
| Three | 1)   Has essential training equipment | Ordinal question | 3.6.1 |
|  | 2)   Has perfect management system | Ordinal question | 3.6.2 |
| Four | 1)   Biosafety, risk evaluation and quality control | Ordinal question | 4.8.1 |
| Five | 1)   Willingness to work on global health | Open question | 5.5.1 |
|  | 2)   Trainee’s evaluation on the Hub | Ordinal question | 5.6.1 |

Additional file 7

**Table 6. The judgment on additional proposed indicators by experts (number, %)**

| **Indicator** | **Round** | **Necessity** | | | | |  | **Feasibility** | | | | |
| --- | --- | --- | --- | --- | --- | --- | --- | --- | --- | --- | --- | --- |
|  |  | **Very good** | **Good** | **Middle** | **Poor** | **Very poor** |  | **Very good** | **Good** | **Middle** | **Poor** | **Very poor** |
| 1.10.1 | 1 | 12 | 6 | 0 | 0 | 0 |  | 9 | 7 | 1 | 0 | 0 |
|  | 2 | 18(22.5) | 44(55.0) | 14(17.5) | 2(2.5) | 0(0.0) |  | 9(11.3) | 45(56.3) | 22(27.5) | 2(2.5) | 0(0.0) |
| 2.6.1 | 1 | 7 | 5 | 1 | 0 | 0 |  | 5 | 7 | 1 | 0 | 0 |
|  | 2 | 40(50.0) | 35(43.8) | 3(3.8) | 0(0.0) | 0(0.0) |  | 37(46.3) | 32(40.0) | 8(10.0) | 1(1.3) | 0(0.0) |
| 3.6.1 | 1 | 3 | 5 | 0 | 0 | 0 |  | 2 | 6 | 0 | 0 | 0 |
|  | 2 | 43(53.8) | 33(41.3) | 2(2.5) | 1(1.3) | 1(1.3) |  | 40(50.0) | 35(43.8) | 2(2.5) | 0(0.0) | 0(0.0) |
| 3.6.2 | 1 | 3 | 1 | 0 | 0 | 0 |  | 2 | 2 | 0 | 0 | 0 |
|  | 2 | 37(46.3) | 40(50.0) | 3(3.8) | 0(0.0) | 0(0.0) |  | 35(43.8) | 43(53.8) | 1(1.3) | 0(0.0) | 0(0.0) |
| 4.8.1 | 1 | 2 | 2 | 1 | 0 | 0 |  | 2 | 3 | 0 | 0 | 0 |
|  | 2 | 39(48.8) | 28(35.0) | 9(11.3) | 0(0.0) | 0(0.0) |  | 28(35.0) | 37(46.3) | 11(13.8) | 0(0.0) | 0(0.0) |
| 5.5.1 | 1 | 4 | 2 | 0 | 0 | 0 |  | 2 | 4 | 0 | 0 | 0 |
|  | 2 | 28(35.0) | 43(53.8) | 4(5.0) | 0(0.0) | 0(0.0) |  | 21(26.3) | 49(61.3) | 5(6.3) | 0(0.0) | 0(0.0) |
| 5.6.1 | 1 | 3 | 2 | 0 | 0 | 0 |  | 2 | 3 | 0 | 0 | 0 |
|  | 2 | 35(43.8) | 41(51.3) | 3(3.8) | 0(0.0) | 0(0.0) |  | 38(47.5) | 38(47.5) | 2(2.5) | 0(0.0) | 0(0.0) |
